# Author Correction: Acidification suppresses the natural capacity of soil microbiome to fight pathogenic *Fusarium* infections

**DOI:** 10.1038/s41467-023-41564-4

**Published:** 2023-10-04

**Authors:** Xiaogang Li, Dele Chen, Víctor J. Carrión, Daniel Revillini, Shan Yin, Yuanhua Dong, Taolin Zhang, Xingxiang Wang, Manuel Delgado-Baquerizo

**Affiliations:** 1 State Key Laboratory of TreeGenetics and Breeding, College of Ecology and Environment, Nanjing Forestry University, Nanjing, China; 2 Co-InnovationCenter for Sustainable Forestry in Southern China, Nanjing Forestry University, Nanjing, China; 3 Key Laboratory of Soil Environment and Pollution Remediation, Institute of Soil Science, Chinese Academy of Sciences, Nanjing, China; 4 School of Agriculture and Biology, Shanghai Jiao Tong University, Shanghai Yangtze River Delta Eco-Environmental Change and ManagementObservation and Research Station, Ministry of Science and Technology, Shanghai, China; 5 Microbial Biotechnology, Institute of Biology, Leiden University, Leiden, the Netherlands; 6 Departamento deMicrobiología, Facultad de Ciencias, Campus Universitario de Teatinos s/n, Universidad de Málaga, Málaga, Spain; 7 Department of Microbial Ecology, Netherlands Institute of Ecology (NIOO-KNAW), Wageningen, The Netherlands; 8 Instituto de Hortofruticultura Subtropical y Mediterránea La Mayora (IHSM) UMA-CSIC, 29010 Málaga, Spain; 9 Laboratorio de Biodiversidad y Funcionamiento Ecosistémico, Instituto de Recursos Naturales y Agrobiología de Sevilla (IRNAS), CSIC, Sevilla, Spain; 10 Ecological Experimental Station of Red Soil, Chinese Academy of Sciences, Yingtan, China

**Keywords:** Agroecology, Agroecology, Parasitism, Plant physiology

Correction to: *Nature Communications* 10.1038/s41467-023-40810-z, published online 22 August 2023

In this article the affiliation ‘Instituto de Hortofruticultura Subtropical y Mediterránea La Mayora (IHSM) UMA-CSIC, 29010 Málaga, Spain’ for Victor Carrión was missing. The original article has been corrected.

