# Supplementary material for: Acidification suppresses the natural capacity of soil microbiome to fight pathogenic Fusarium infections
Source: Nat Commun. 2023 Aug 22;14:5090. doi: 10.1038/s41467-023-40810-z (PMC10444831; doi:10.1038/s41467-023-40810-z)
Supplement: Supplementary file 1 — Supplementary Information [file 41467_2023_40810_MOESM1_ESM.pdf]

Acidification suppresses the natural capacity of soil microbiome to fight pathogenic *Fusarium* infections

Xiaogang Li[1, 3, #], Dele Chen[2, 4, #], Víctor J. Carrión[5], Daniel Revillini[6], Shan Yin[4], Yuanhua Dong[2], Taolin Zhang[2], Xingxiang Wang[2,7]*, Manuel Delgado-Baquerizo[6]*

[1]State Key Laboratory of Tree Genetics and Breeding, Nanjing Forestry University, Nanjing 210037, China

[2]Key Laboratory of Soil Environment and Pollution Remediation, Institute of Soil Science, Chinese Academy of Sciences, Nanjing 210008, China

[3]Co-Innovation Center for Sustainable Forestry in Southern China, Nanjing Forestry University, Nanjing, China

[4]School of Agriculture and Biology, Shanghai Jiao Tong University, Shanghai Yangtze River Delta Eco-Environmental Change and Management Observation and Research Station, Ministry of Science and Technology, Shanghai 200240, China

[5]Microbial Biotechnology, Institute of Biology, Leiden University, Leiden, the Netherlands

[6]Laboratorio de Biodiversidad y Funcionamiento Ecosistémico, Instituto de Recursos Naturales y Agrobiología de Sevilla (IRNAS), CSIC, Sevilla, Spain

[7]Ecological Experimental Station of Red Soil, Chinese Academy of Sciences, Yingtan 335211, China

[#]These authors contributed equally to this work.

***Corresponding authors**

E-mail address: xxwang@issas.ac.cn (X. Wang); m.delgadobaquerizo@gmail.com (Manuel Delgado-Baquerizo)

Supplementary Figures

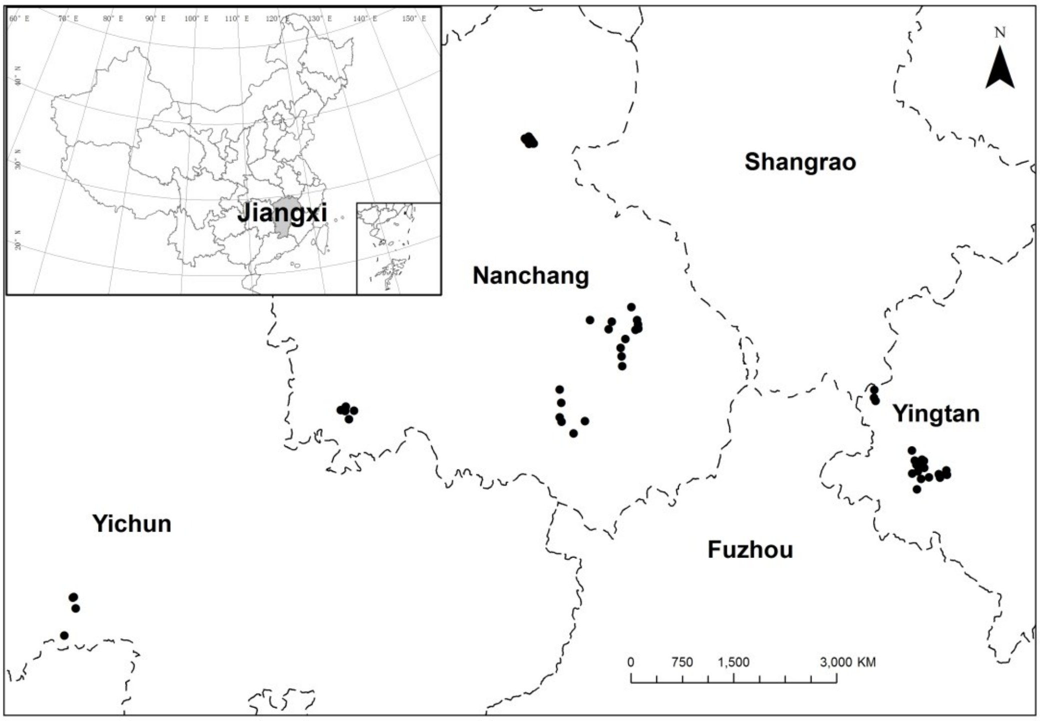

Supplementary Figure 1. Geographical distribution of sampling fields across southeast China (*n* = 60 study fields). Yichun, Nanchang, Fuzhou, Shangrao, and Yingtan are cities in southeast China. Dots denote the field locations of paired plant and soil sampling.

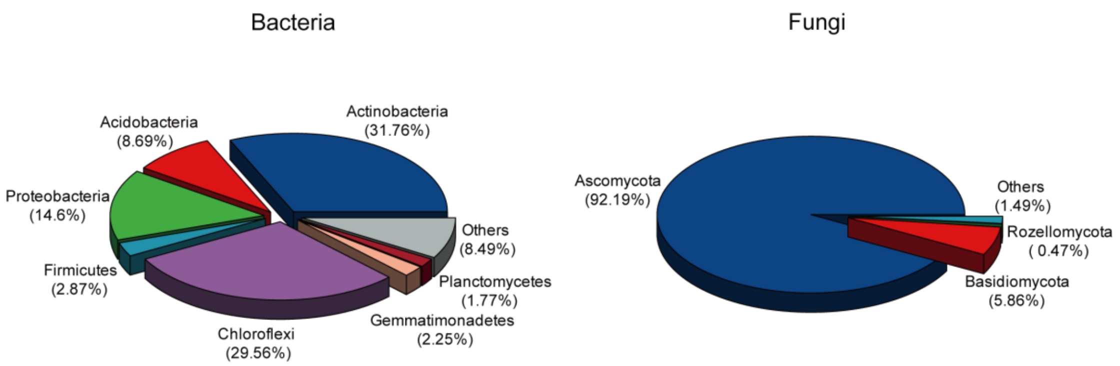

Supplementary Figure 2. Compositional traits of soil bacteria and fungi at the phylum level via Illumina sequencing. Values in brackets denote the percentage of the corresponding phylum.

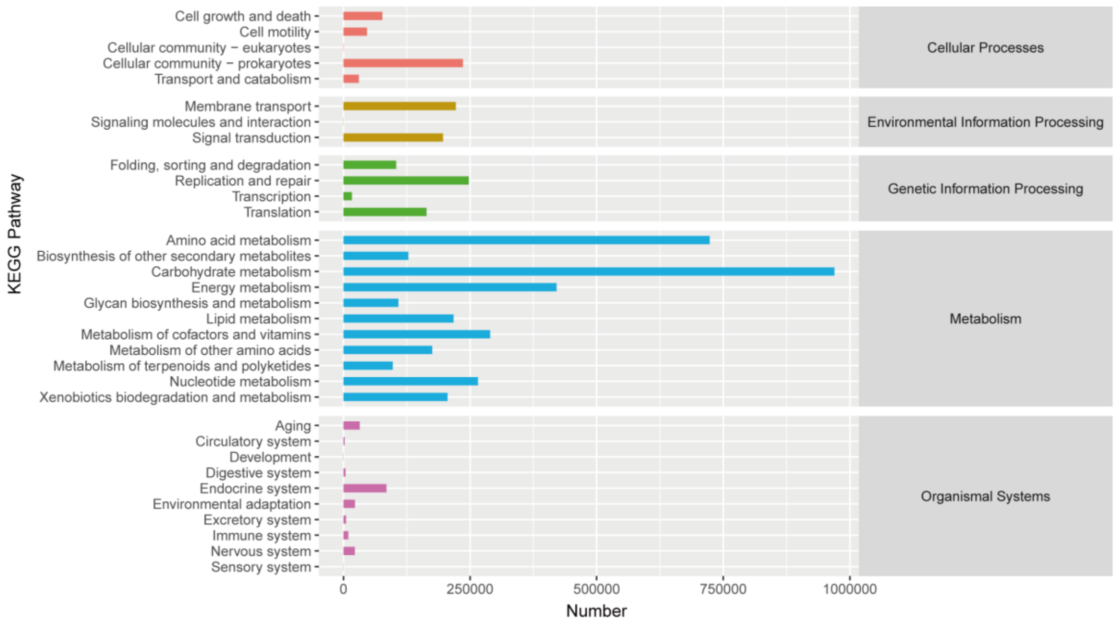

Supplementary Figure 3. Main KEGG pathways for gene functional annotation. The horizontal coordinate represents the number of proteins annotated to the respective metabolic pathways, while the longitudinal coordinate corresponds to various second-level metabolic pathways in the KEGG database. The first-level classification of each metabolic pathway is listed in the right column.

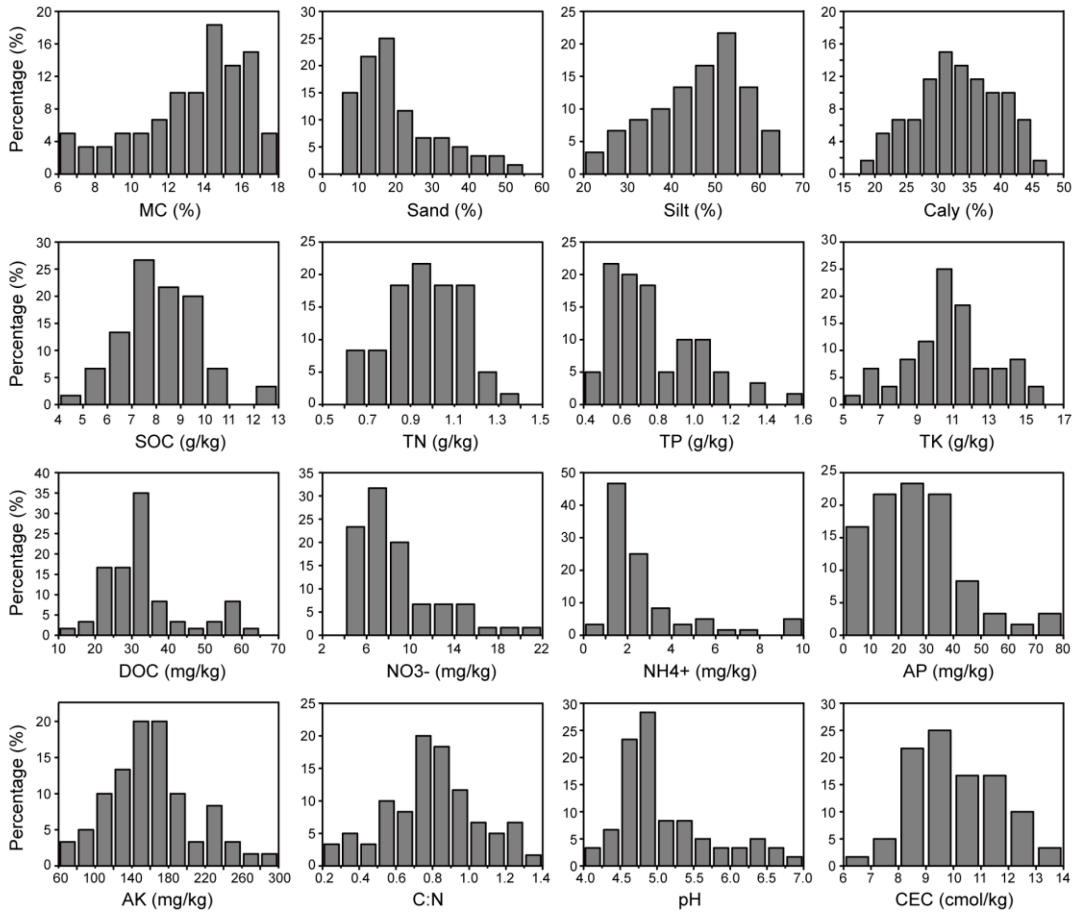

Supplementary Figure 4. Frequency distributions of soil physiochemical properties. MC, moisture content; SOC, soil organic carbon; TN, total nitrogen; TP, total phosphorus; TK, total potassium; DOC, soil dissolved organic carbon; NO_3^-, nitrate; NH_4^+, ammonium; AP, available phosphorus; AK, available potassium; C:N, SOC/(nitrate + ammonium); CEC, cation exchange capacity. The horizontal coordinate represents the content of physicochemical factors, while the longitudinal coordinate represents the percentage of samples in the total number of samples corresponding to the content of the respective physicochemical factor.

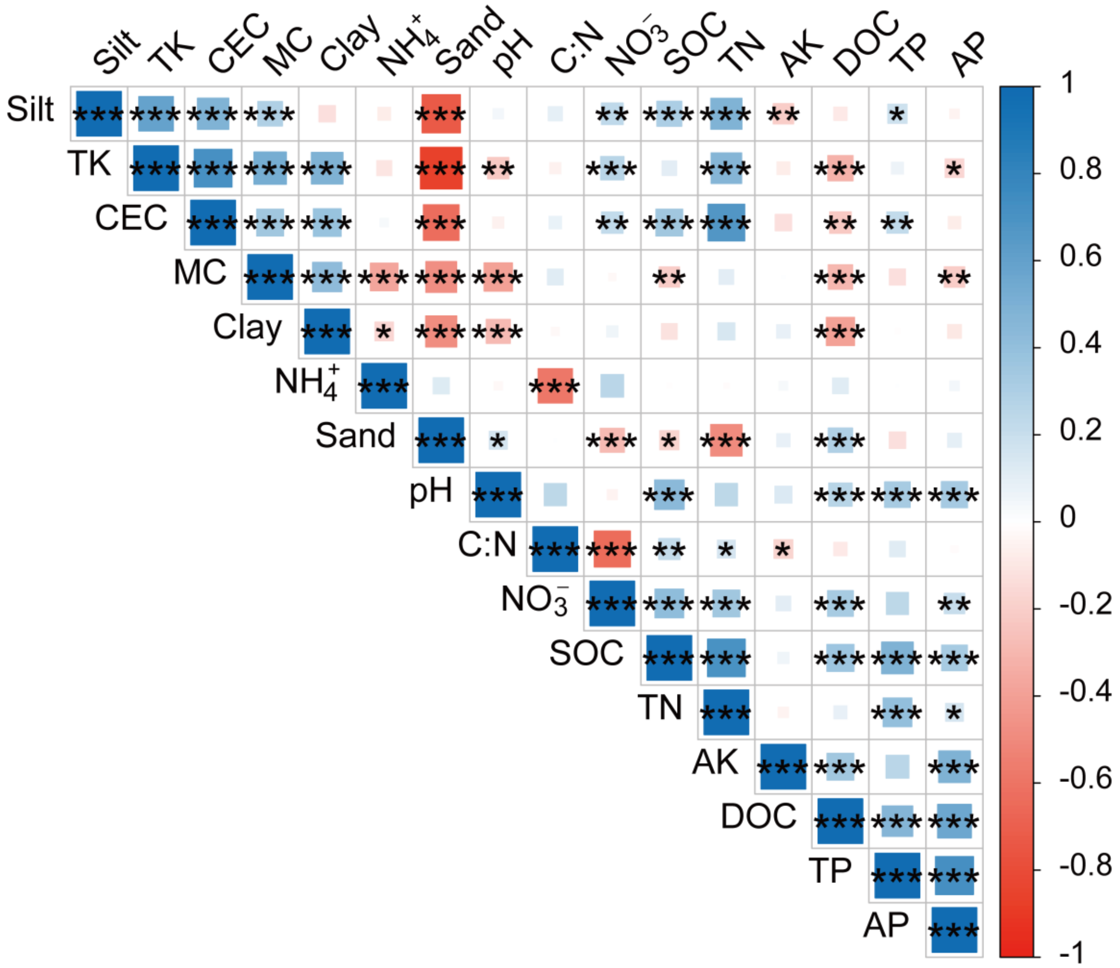

Supplementary Figure 5. Correlations among soil physiochemical properties. Spearman correlations between soil physiochemical properties are noted with colored squares. Blue colors indicate positive correlation, and red colors indicate negative correlation. *P* values were adjusted by Benjamini–Hochberg false discovery correction (*$P < 0.05$, **$P < 0.01$, ***$P < 0.001$; $n = 180$). Information on environmental factors included in the analysis can be found in Fig. S4.

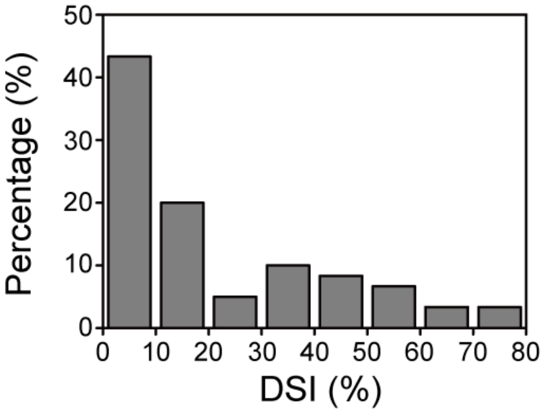

Supplementary Figure 6. Frequency distribution of disease severity index (DSI). The horizontal coordinate represents the disease severity index according to the disease severity calculation method in the Supplementary Text, while the longitudinal coordinate represents the percentage of number of surveyed fields corresponding to the DSI in the total number of fields.

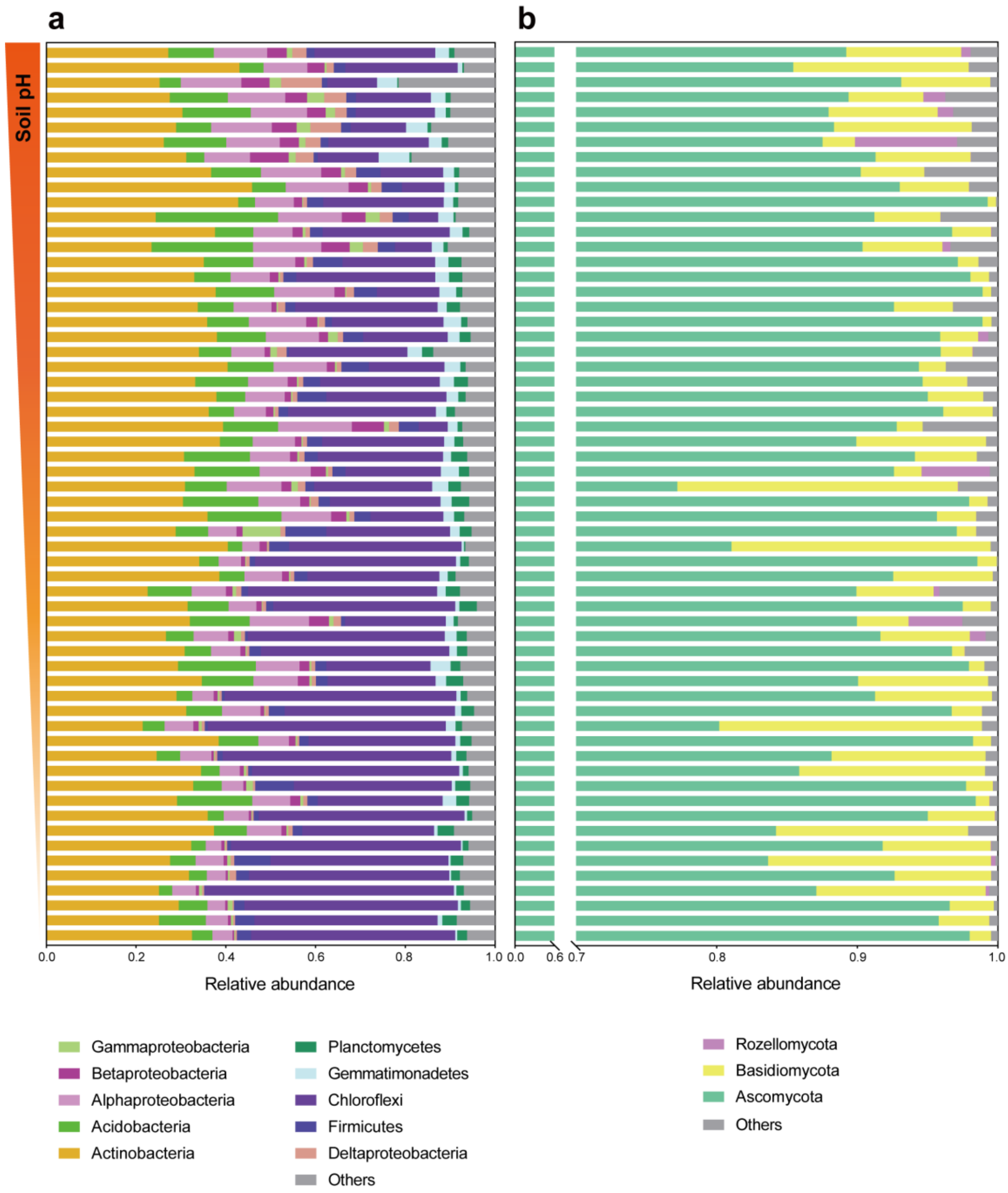

Supplementary Figure 7. Relative abundance at the phylum level of bacterial (a) and fungal (b) communities from soil samples of different pH. The horizontal coordinate represents the relative abundances of bacterial and fungal taxa at the phylum level, while the longitudinal coordinate represents soil pH from 4 to 7 of 60 peanut fields.

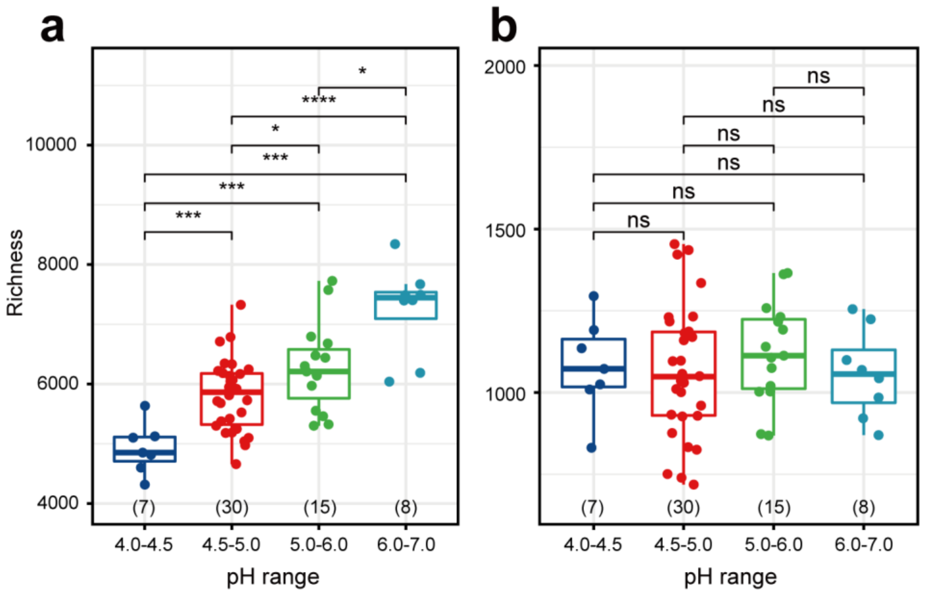

Supplementary Figure 8. Effects of soil acidification on Chao1 richness of the bacterial (a) and fungal (b) communities from soil samples of different pH ranges (*n* = 60). Boxplots indicate median (box center line), 25th, 75th percentiles (box), and 5th and 95th percentiles (whiskers). Asterisks indicate significant differences as represented by the Wilcoxon test (two-sided, $*P < 0.05$, $**P < 0.01$, $***P < 0.001$), and "ns" means not significant difference. Numbers in brackets denote the sample size in corresponding boxplot.

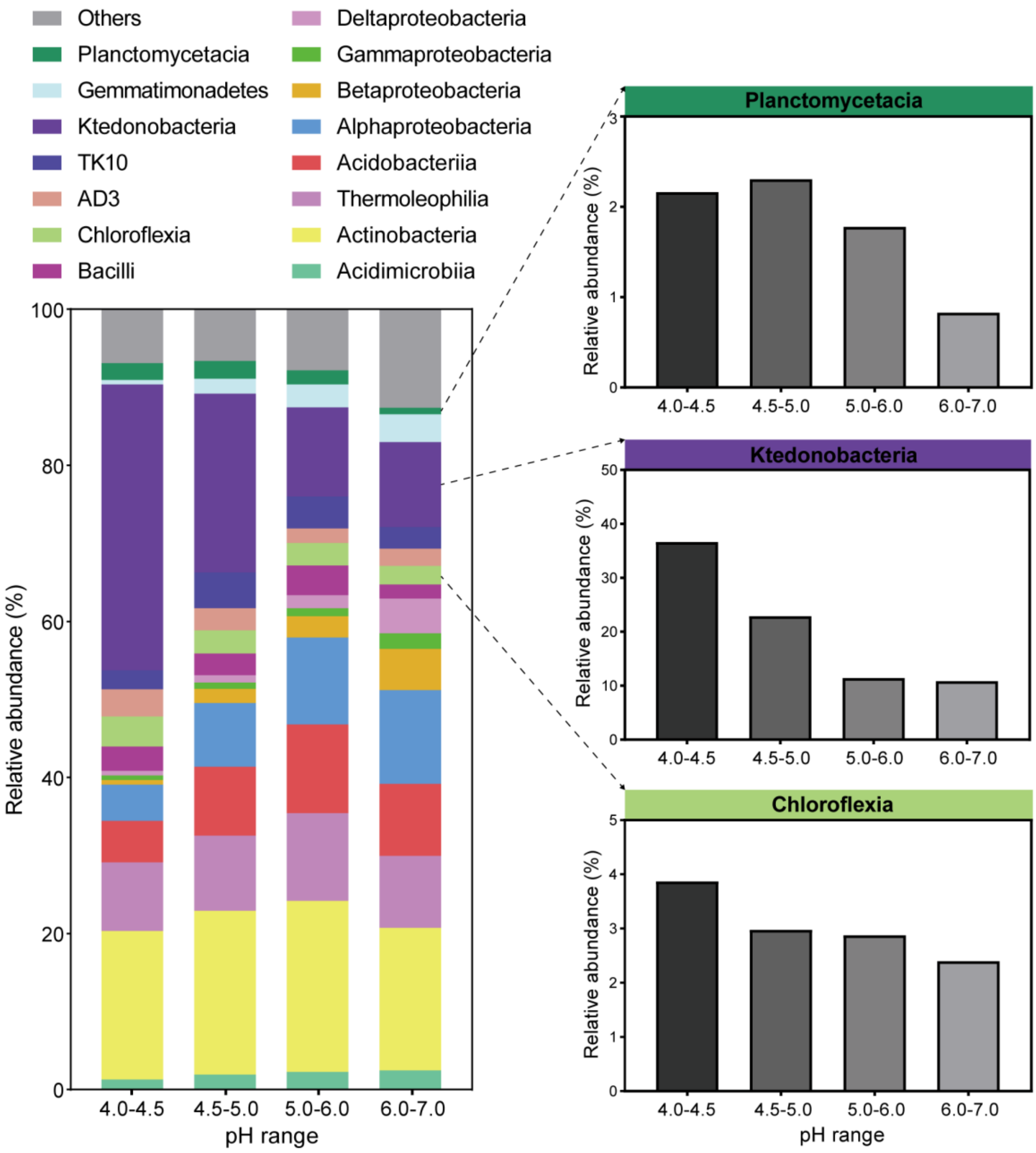

Supplementary Figure 9. Compositional traits of soil bacteria at the class level categorized by different pH ranges. The arrows refer to the relative abundances corresponding to the three classes, Planctomycetacia, Ktedonobacteria and Chloroflexia, in the four pH ranges. These three highly abundant classes were enriched under soil acidification.

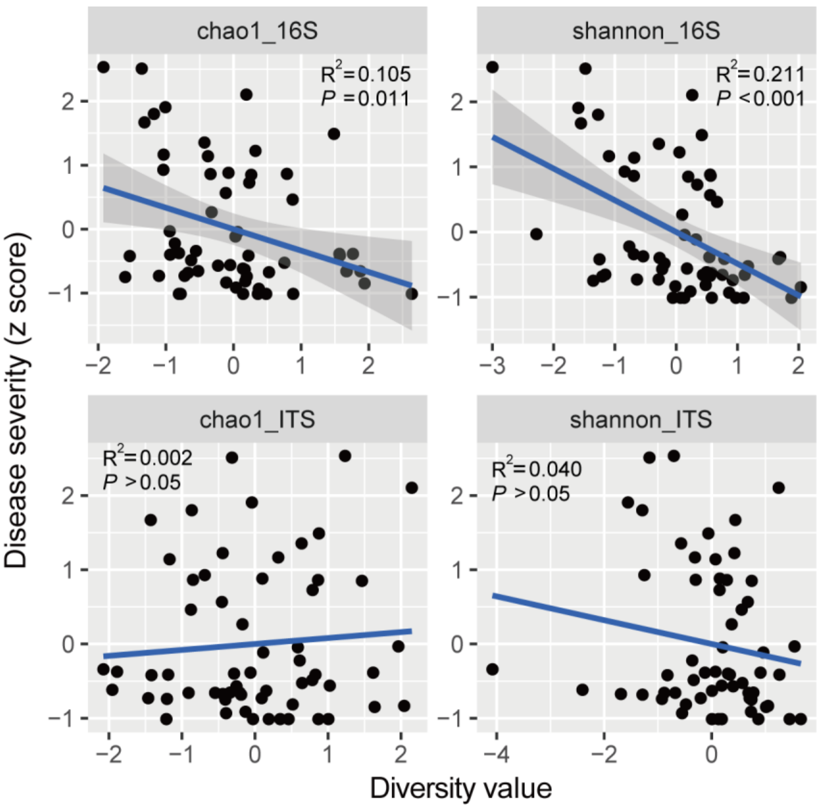

Supplementary Figure 10. Ordinary least squares linear regression between the disease severity index and microbial diversity (n = 60). The statistical test used is F-test based on two-sided tests, and $P < 0.05$ denotes the overall significance of the regression model. The blue fitted lines are regression lines from OLS linear regression, and the shaded areas indicate the 95% confidence interval of the regression fit.

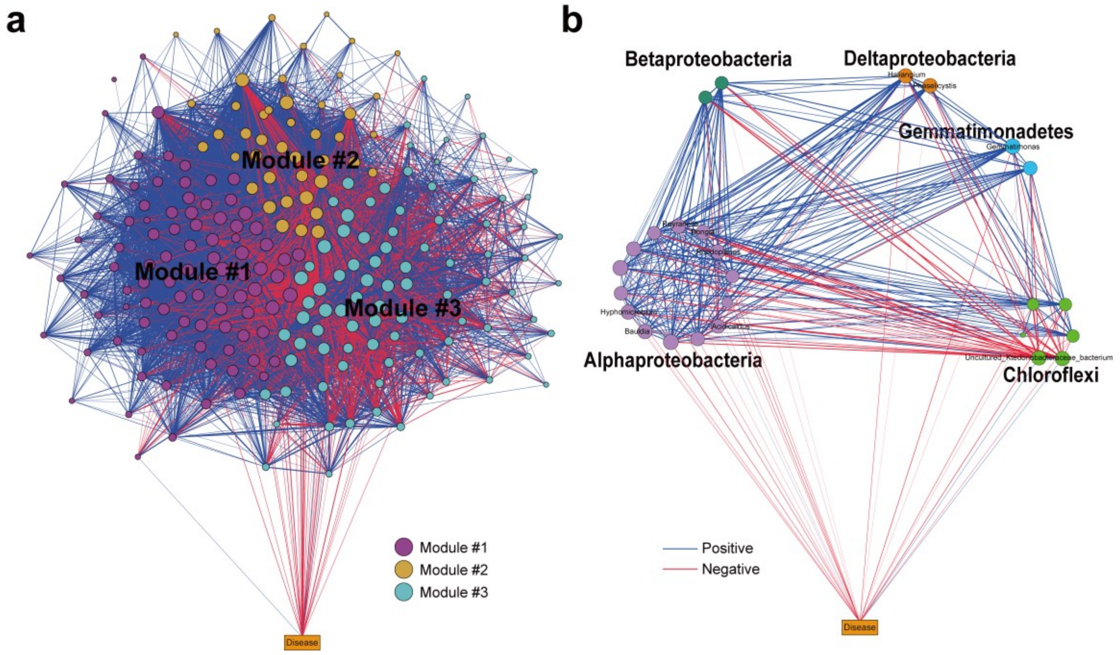

Supplementary Figure 11. Co-occurrence network and keystone taxa that reveal responses to disease severity. a Network diagram with nodes colored according to each of the three main ecological clusters (modules #1, 2 and 3). The genera within the five bacterial phyla (*i.e.*, significant predictors) among all the samples were chosen for network analysis to assess the bacterial interactive effects on disease severity. The co-occurrence patterns were constructed based on Spearman correlation matrix. Only those with a cut-off at an absolute $r > 0.3$ and a $P < 0.05$, were considered statistically robust between taxa and retained. b Keystone genera that reveal significant correlations with disease severity. Among them, 13 genera in Alphaproteobacteria, 2 genera in Betaproteobacteria, 2 genera in Deltaproteobacteria, 2 genera in Gemmatimonadetes, and 4 genera in Chloroflexi were identified as negatively correlated with disease severity, whereas 1 genus in Alphaproteobacteria and 2 genera in Chloroflexi were identified as positively correlated with disease severity. The thickness of each connection between two nodes is proportional to the value of correlation coefficients.

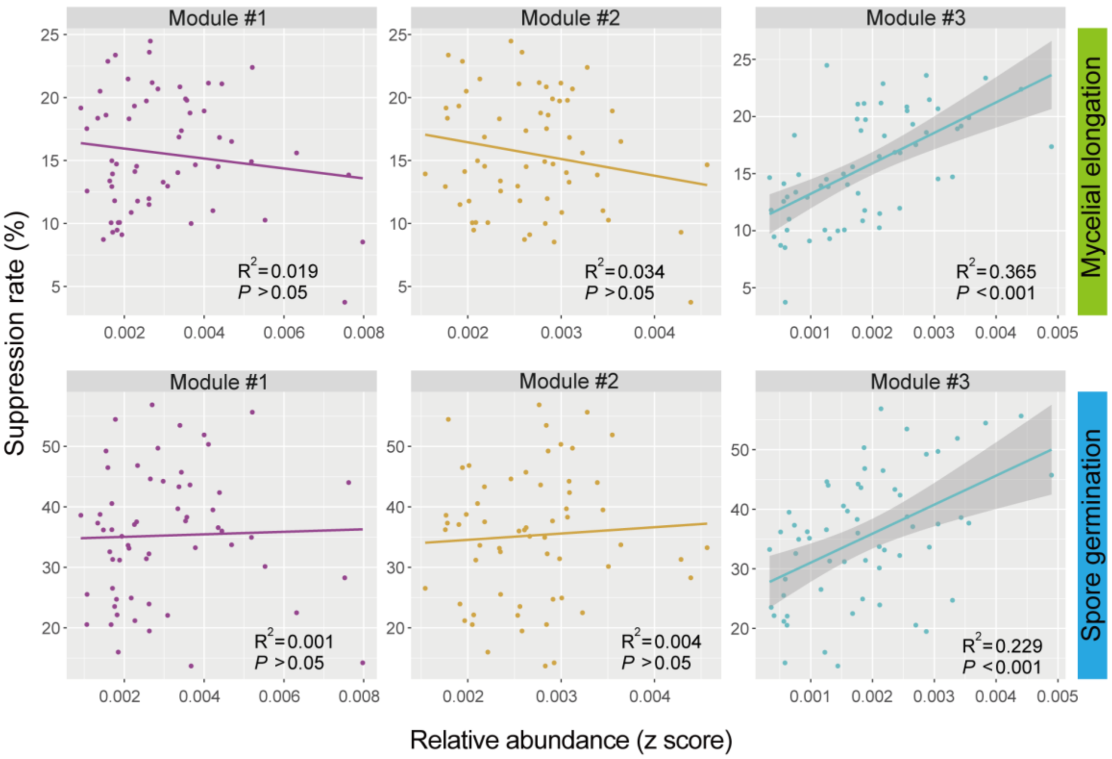

Supplementary Figure 12. Ordinary least squares linear regression between the suppression rate (%) of mycelial elongation and spore germination and the average relative abundance of the main ecological clustering modules (*n* = 60). The statistical test used is *F*-test based on two-sided tests. $P > 0.05$ denotes the overall not significance of the regression model, and $P < 0.001$ denotes the overall significance of the regression model. The blue fitted lines are regression lines from OLS linear regression, and the shaded areas indicate the 95% confidence interval of the regression fit.

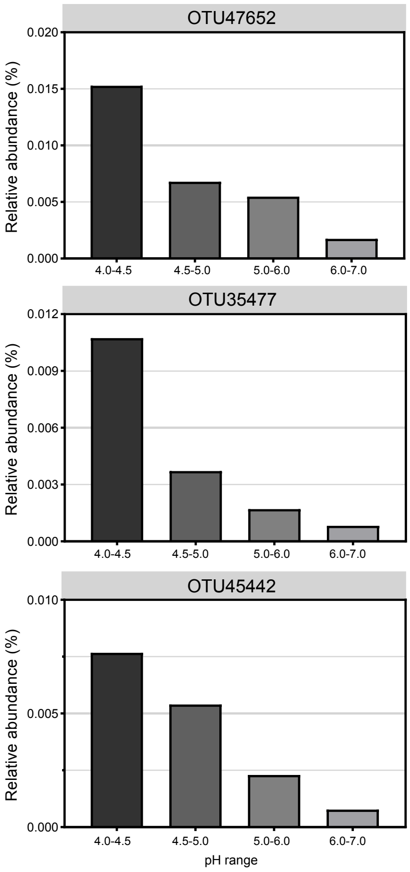

Supplementary Figure 13. Relative abundances of pathogenic fungi categorized by different pH ranges via Illumina sequencing. OTU47652 belongs to *Fusarium solani*, OTU35477 and OTU45442 belong to *Fusarium falciforme*.

Supplementary Tables

Supplementary Table 1. Primer sequences used for bacterial 16S rRNA and fungal ITS high-throughput sequencing.

Target group	Primer name and sequence (5´-3´)	Reference
Bacteria	F338, ACTCCTACGGGAGGCAGCA	Derakhshani et al., 2016
	R806, GGACTACHVGGGTWTCTAAT	
Fungi	ITS3, GCATCGATGAAGAACGCAGC	Orgiazzi et al., 2012
	ITS4, TCCTCCGCTTATTGATATGC	

Supplementary Table 2. Sequences of oligonucleotide primers required for quantitative PCR.

Target group	Primer name and sequence (5´-3´)	Approximate size of product (bp)	Annealing temp (°C)	Reference
Bacteria	Eub338F, ACTCCTACGGGAGGCAGCAG	200	53	Fierer et al., 2005
	Eub518R, ATTACCGCGGCTGCTGG			
Fungi	ITS1f, TCC GTA GGT GAA CCT GCG G	300	53	
	5.8s, CGC TGC GTT CTT CAT CG			

Supplementary Table 3. Composition of growth media used in this study.

Media	Composition
PDB	Potato Dextrose (Sigma-Aldrich, St. Louis, Missouri, USA) 38 g L^{-1}
NA	Beef Extract (OXOID, Hampshire, England) 3 g L^{-1}, Soybean Peptone (OXOID) 10 g L^{-1}, Sodium Chloride (Sigma-Aldrich) 5 g L^{-1}, Agar Powder CMN (Boom, Meppel, Netherlands) 20 g L^{-1}

Supplementary Table 4. Summary of the shotgun metagenomic sequencing raw data.

Sample	Reads	Bases (bp)	N (%)	GC (%)	Q20 (%)	Q30 (%)
A1	72734028	10982838228	0.00080	60.38	91.59	81.13
A2	73031166	11027706066	0.00094	58.98	91.93	81.80
A3	72962386	11017320286	0.00151	61.07	90.96	80.25
A4	80064940	12089805940	0.00161	60.85	91.52	81.35
B1	82205096	12412969496	0.00164	61.63	91.42	81.24
B2	71974886	10868207786	0.00191	59.79	91.94	82.27
B3	74668566	11274953466	0.00186	60.98	91.79	82.00
B4	74872542	11305753842	0.00196	61.03	92.14	82.75
C1	75878748	11457690948	0.00172	62.35	91.31	81.14
C2	74119900	11192104900	0.00154	61.72	91.00	80.38
C3	74457856	11243136256	0.00179	61.58	91.23	80.97
C4	72327326	10921426226	0.00148	61.29	91.20	80.91

Note: A1-A4 represent soil communities at pH of 4.0-4.5; B1-B4 represent soil communities at pH of 4.5-5.0; C1-C4 represent soil communities at pH of 5.0-6.0. N (%) is the proportion of fuzzy bases in the total number of bases; GC (%) is the GC content, that is, the proportion of G base and C base in the total number of bases; Q20 (%) and Q30 (%) are the proportion of bases whose determination accuracy is 99% and 99.9%, respectively.

Supplementary Table 5. Contigs of the genome assembly via metagenomic sequencing.

Sample	Min. sequence length (bp)	Max. sequence length (bp)	N50 length (bp)	Sequence number
A1	200	166785	672	148842
A2	200	118226	571	120715
A3	200	196856	681	147281
A4	200	472709	611	136881
B1	200	79032	545	108887
B2	200	169252	653	117582
B3	200	190028	576	114465
B4	200	118880	520	86558
C1	200	68387	479	61327
C2	200	517262	542	89173
C3	200	71629	519	79660
C4	200	60947	495	69514

Note: N50 length refers to the corresponding length of the last sequence when the length of the sum reaches 50% of the total length of contig sequences in order from long to short. Sequence number refers to those sequence longer than 1 kb. Information on A1-C4 can be found in Table S4.

Supplementary Table 6. Mantel test results of soil properties on bacterial and fungal communities.

	Community dissimilarity	
	Bacteria	Fungi
pH	0.419***	-0.020
MC	0.160*	0.012
SOC	0.338***	-0.036
DOC	-0.041	-0.005
TN	0.128*	0.018
NO_3^-	0.105*	0.005
NH_4^+	0.042	-0.018
C:N	0.028	0.022
TP	-0.014	0.021
AP	-0.042	-0.031
TK	-0.053	0.067
AK	-0.118	-0.041
CEC	-0.048	0.028
Sand	-0.065	0.010
Silt	0.098*	-0.005
Clay	0.271***	-0.038

Abbreviations: MC, moisture content (%); SOC, soil organic carbon (g kg^{-1}); DOC, dissolved organic carbon (mg kg^{-1}); TN, total nitrogen (g kg^{-1}); NO_3^-, soil nitrate (mg kg^{-1}); NH_4^+, soil ammonium (mg kg^{-1}); C:N, carbon to nitrogen ratio; TP, total phosphorus (g kg^{-1}); AP, available phosphorus (mg kg^{-1}); TK, total potassium (g kg^{-1}); AK, available potassium (mg kg^{-1}); CEC, cation exchange capacity (cmol kg^{-1}). The statistical tests are two-sided, and significant effects are indicated by *** $P < 0.001$, ** $P < 0.01$, * $P < 0.05$.

Supplementary Table 7. Comparison of the relative abundances of the dominant taxa among different pH ranges. Values are means (SD). Values at the same rows followed by different letters differed significantly at *P* < 0.05 based on Duncan's multiple comparisons test.

Bacteria/Phylum level	pH			
	4.0-4.5	4.5-5.0	5.0-6.0	6.0-7.0
Actinobacteria	0.29(0.03)b	0.33(0.05)ab	0.35(0.06)a	0.30(0.06)b
Acidobacteria	0.05(0.03)b	0.09(0.04)ab	0.11(0.06)a	0.09(0.04)ab
Alphaproteobacteria	0.05(0.01)c	0.08(0.03)b	0.11(0.03)a	0.12(0.01)a
Betaproteobacteria	0.01(0.00)c	0.02(0.01)b	0.03(0.02)b	0.05(0.02)a
Gammaproteobacteria	0.01(0.00)b	0.01(0.01)b	0.01(0.01)ab	0.02(0.01)a
Deltaproteobacteria	0.01(0.00)c	0.01(0.00)bc	0.02(0.01)b	0.04(0.02)a
Firmicutes	0.03(0.03)ab	0.03(0.02)ab	0.04(0.02)a	0.02(0.02)b
Chloroflexi	0.46(0.06)a	0.33(0.11)b	0.20(0.08)c	0.18(0.06)c
Gemmatimonadetes	0.01(0.00)c	0.02(0.01)b	0.03(0.01)a	0.04(0.02)a
Planctomycetes	0.02(0.01)a	0.02(0.01)a	0.02(0.01)a	0.01(0.00)b
Others	0.07(0.01)b	0.07(0.01)b	0.08(0.02)b	0.13(0.05)a

Fungi/Phylum level	pH			
	4.0-4.5	4.5-5.0	5.0-6.0	6.0-7.0
Ascomycota	0.92(0.05)ab	0.92(0.06)ab	0.95(0.03)a	0.89(0.02)b
Basidiomycota	0.07(0.05)a	0.06(0.06)ab	0.03(0.02)b	0.07(0.03)a
Rozellomycota	0.001(0.001)b	0.003(0.011)ab	0.001(0.002)b	0.013(0.025)a
Others	0.004(0.002)b	0.013(0.012)ab	0.020(0.016)a	0.023(0.010)a

Supplementary Table 8. Bacterial and fungal biomarkers sensitive to disease severity revealed by co-occurrence analysis.

Genus	Family	Order	Class	Phylum
Gemmatimonas	Gemmatimonadaceae	Gemmatimonadales	Gemmatimonadetes	Gemmatimonadetes
Uncultured_Gemmatimonadaceae_bacterium	Gemmatimonadaceae	Gemmatimonadales	Gemmatimonadetes	Gemmatimonadetes
Acidicaldus	Acetobacteraceae	Acetobacterales	Alphaproteobacteria	Proteobacteria
Dongia	Dongiaceae	Dongiales	Alphaproteobacteria	Proteobacteria
Uncultured_Elsteraceae_bacterium	Elsteraceae	Elsterales	Alphaproteobacteria	Proteobacteria
Uncultured_Micropepsaceae_bacterium	Micropepsaceae	Micropepsales	Alphaproteobacteria	Proteobacteria
Reyranella	Reyranellaceae	Reyranellales	Alphaproteobacteria	Proteobacteria
Unidentified_Devosiaceae_bacterium	Devosiaceae	Rhizobiales	Alphaproteobacteria	Proteobacteria
Hyphomicrobium	Hyphomicrobiaceae	Rhizobiales	Alphaproteobacteria	Proteobacteria
Pedomicrobium	Hyphomicrobiaceae	Rhizobiales	Alphaproteobacteria	Proteobacteria
Bauldia	Rhizobiales_Incertae_Sedis	Rhizobiales	Alphaproteobacteria	Proteobacteria
Nordella	Rhizobiales_Incertae_Sedis	Rhizobiales	Alphaproteobacteria	Proteobacteria
Bradyrhizobium	Xanthobacteraceae	Rhizobiales	Alphaproteobacteria	Proteobacteria
Rhodoplanes	Xanthobacteraceae	Rhizobiales	Alphaproteobacteria	Proteobacteria

Genus	Family	Order	Class	Phylum
Uncultured_Xanthobacteraceae_bacterium	Xanthobacteraceae	Rhizobiales	Alphaproteobacteria	Proteobacteria
Unidentified_Xanthobacteraceae_bacterium	Xanthobacteraceae	Rhizobiales	Alphaproteobacteria	Proteobacteria
Ellin6067	Nitrosomonadaceae	Betaproteobacteriales	Betaproteobacteria	Proteobacteria
Uncultured_SC-I-84_bacterium	SC-I-84	Betaproteobacteriales	Betaproteobacteria	Proteobacteria
Haliangium	Haliangiaceae	Myxococcales	Deltaproteobacteria	Proteobacteria
Phaselicystis	Phaselicystidaceae	Myxococcales	Deltaproteobacteria	Proteobacteria
Uncultured_C0119_bacterium	Uncultured_bacterium	C0119	Ktedonobacteria	Chloroflexi
1921-3	Ktedonobacteraceae	Ktedonobacterales	Ktedonobacteria	Chloroflexi
Uncultured_Ktedonobacteraceae_bacterium	Ktedonobacteraceae	Ktedonobacterales	Ktedonobacteria	Chloroflexi
Uncultured_A4b_bacterium	A4b	SBR1031	Anaerolineae	Chloroflexi
Uncultured_Roseiflexaceae_bacterium	Roseiflexaceae	Chloroflexales	Chloroflexia	Chloroflexi
Uncultured_KD4-96_bacterium	Uncultured_bacterium	Uncultured_bacterium	KD4-96	Chloroflexi

Note: Those genera in bold were recognized as the keystone taxa based on the highest degree, highest closeness centrality, and lowest betweenness centrality in the network (Banerjee et al., 2018).

Supplementary Table 9. Role of key taxa contributing to disease severity based on Spearman’s correlation.

Genus	r
Gemmatimonas	-0.47
Uncultured_Gemmatimonadaceae_bacterium	-0.31
Acidicaldus	0.33
Dongia	-0.41
Uncultured_Elsteraceae_bacterium	-0.36
Uncultured_Micropepsaceae_bacterium	-0.37
Reyranella	-0.32
Unidentified_Devosiaceae_bacterium	-0.36
Hyphomicrobium	-0.38
Pedomicrobium	-0.40
Bauldia	-0.30
Nordella	-0.35
Bradyrhizobium	-0.33
Rhodoplanes	-0.41
Uncultured_Xanthobacteraceae_bacterium	-0.35
Unidentified_Xanthobacteraceae_bacterium	-0.34
Ellin6067	-0.31
Uncultured_SC-I-84_bacterium	-0.32
Haliangium	-0.44
Phaselicystis	-0.31
Uncultured_C0119_bacterium	-0.32
1921-3	0.35
Uncultured_Ktedonobacteraceae_bacterium	0.34
Uncultured_A4b_bacterium	-0.36
Uncultured_Roseiflexaceae_bacterium	-0.40
Uncultured_KD4-96_bacterium	-0.48

Supplementary Table 10. Topological properties of co-occurring bacterial network.

Topological properties	Original network [a]	Simplified network [b]
Number of nodes	246	27
Number of edges	10900	318
Numbers of positive correlations	8649	230
Numbers of negative correlations	2251	88
Average path length	1.701	1.094
Graph density	0.359	0.906
Network diameter	4	2
Average clustering coefficient	0.671	0.938
Average degrees	88.259	23.556
Modularity	0.151	0.048

a. The original network refers to the co-occurrence network in Fig. S11a.

b. The simplified network refers to the co-occurrence network in Fig. S11b.

Supplementary Table 11. Summary of the clean dataset after screening and filtering via metagenomic sequencing.

Sample	HQ Data (bp)	HQ Data (%)	HQ Reads (%)
A1	9932474518	90.44	97.31
A2	10019150422	90.85	97.42
A3	9867841588	89.57	97.30
A4	10923377380	90.35	97.56
B1	11193605113	90.18	97.39
B2	9852857895	90.66	97.59
B3	10206336507	90.52	97.51
B4	10280452897	90.93	97.66
C1	10296894679	89.87	97.37
C2	10023144461	89.56	97.25
C3	10095082166	89.79	97.28
C4	9806651014	89.79	97.31

Note: HQ Data (bp) refers to the total number of bases of high-quality sequences; HQ Data (%) refers to the proportion of the total number of bases of high-quality sequence in the total number of bases of the raw sequence. HQ Reads (%) refers to the proportion of the number of high-quality sequences in the original number of raw sequences. Information on A1-C4 can be found in Table S4.

Supplementary Table 12. Identified volatile compounds produced by bacterial communities.

Compound	*RT*	pH 4.0-4.5	pH 4.5-5.0	pH 5.0-6.0
**Dimethyl sulfide**	2.65	9.31	7.78	6.96
Trichloromethane	3.06	13.83	ND	ND
**Dimethyl disulfide**	3.77	0.68	30.58	31.23
Allomatrine	3.79	4.12	ND	ND
3-Methylenecyclohexene	4.28	0.69	ND	ND
6-Methylchromone-2-carboxylic acid	4.34	0.55	ND	ND
**Dimethyl sulfoxide (DMSO)**	5.59	0.59	0.57	0.39
**Tiapride**	5.64	ND	ND	0.40
1,3-dimethyl benzene	6.04	3.99	ND	1.24
Ethylbenzene	6.05	ND	1.94	0.54
Bicyclo[4.2.0]octa-1,3,5-triene	6.68	30.50	ND	ND
Styrene	6.69	ND	24.89	23.03
2,2,4,6,6-pentamethyl heptane	8.92	0.62	ND	ND
Octamethyl cyclotetrasiloxane	9.03	1.34	0.51	0.43
4-Methyl cycloheptanone	9.19	ND	ND	1.59
1-Methyl-2-(4-methylpentyl) cyclopentane	9.23	1.58	ND	ND
2,5-Dimethylcyclohexanone	9.58	ND	3.21	0.73
o-Cymene	9.67	ND	1.44	0.99
1-Methyl-2-(1-methylethyl)-Benzene	9.71	2.12	ND	ND
2,4,4,6,6,8,8-Heptamethyl-1-nonene	10.35	0.80	ND	ND
2-Methyl-1-undecanol	11.06	ND	ND	3.93
2-Nonanone	11.07	ND	3.63	ND
1-Methyl-4-(1-methylethenyl)-Benzene	11.08	0.66	ND	ND
Decamethyl cyclopentasiloxane	12.31	1.15	5.37	6.68

Compound	*RT*	pH 4.0-4.5	pH 4.5-5.0	pH 5.0-6.0
Dodecamethyl cyclohexasiloxane	15.61	10.85	9.35	10.18
Tetradecamethyl cycloheptasiloxane	18.59	6.26	4.18	4.50
Hexadecamethyl cyclooctasiloxane	21.19	2.58	1.86	1.83
Octadecamethyl cyclononasiloxane	23.28	1.02	1.17	1.11

Note: Shown are those volatiles that were emitted from the bacterial communities extracted from soils with pH of 4.0-4.5, 4.5-5.0, and 5.0-6.0, excluding volatiles that were also present in the controls. Compounds were identified based on retention index and mass spectra. Those volatiles in bold were recognized as sulfurous volatile compounds produced by bacterial communities. RT: retention time. ND: not detected.
