# Supplementary material for: Acidification suppresses the natural capacity of soil microbiome to fight pathogenic Fusarium infections
Source: Nat Commun. 2023 Aug 22;14:5090. doi: 10.1038/s41467-023-40810-z (PMC10444831; doi:10.1038/s41467-023-40810-z)
Supplement: Supplementary file 2 — Reporting Summary [file 41467_2023_40810_MOESM2_ESM.pdf]

nature portfolio

Corresponding author(s): Xingxiang Wang, Manuel Delgado-Baqueriz
Last updated by author(s): Jul 31, 2023

Reporting Summary

Nature Portfolio wishes to improve the reproducibility of the work that we publish. This form provides structure for consistency and transparency in reporting. For further information on Nature Portfolio policies, see our Editorial Policies and the Editorial Policy Checklist.

Statistics

For all statistical analyses, confirm that the following items are present in the figure legend, table legend, main text, or Methods section.

n/a	Confirmed	
☐	☒	The exact sample size (*n*) for each experimental group/condition, given as a discrete number and unit of measurement
☐	☒	A statement on whether measurements were taken from distinct samples or whether the same sample was measured repeatedly
☐	☒	The statistical test(s) used AND whether they are one- or two-sided<br>*Only common tests should be described solely by name; describe more complex techniques in the Methods section.*
☐	☒	A description of all covariates tested
☐	☒	A description of any assumptions or corrections, such as tests of normality and adjustment for multiple comparisons
☐	☒	A full description of the statistical parameters including central tendency (e.g. means) or other basic estimates (e.g. regression coefficient) AND variation (e.g. standard deviation) or associated estimates of uncertainty (e.g. confidence intervals)
☐	☒	For null hypothesis testing, the test statistic (e.g. *F*, *t*, *r*) with confidence intervals, effect sizes, degrees of freedom and *P* value noted<br>*Give P values as exact values whenever suitable.*
☒	☐	For Bayesian analysis, information on the choice of priors and Markov chain Monte Carlo settings
☒	☐	For hierarchical and complex designs, identification of the appropriate level for tests and full reporting of outcomes
☐	☒	Estimates of effect sizes (e.g. Cohen's *d*, Pearson's *r*), indicating how they were calculated

Our web collection on statistics for biologists contains articles on many of the points above.

Software and code

Policy information about availability of computer code

Data collection	No software was used for data collection.
Data analysis	For Bioinformatic analysis, a combination of QIIME(v2), SeqPrep (v1.3.2), VSEARCH (v1.4.0), UPARSE (v7.1), SILVA (v123), UNITE (v7.1), and KEGG (Release 79.0) were used. The rest of the analysis in this study were made with vegan, pheatmap, ggplot2, igraph, Hmisc, randomForest and rfPermute packages (https://cran.r-project.org) in R 3.6.1.

For manuscripts utilizing custom algorithms or software that are central to the research but not yet described in published literature, software must be made available to editors and reviewers. We strongly encourage code deposition in a community repository (e.g. GitHub). See the Nature Portfolio guidelines for submitting code & software for further information.

Data

Policy information about availability of data

All manuscripts must include a data availability statement. This statement should provide the following information, where applicable:
- Accession codes, unique identifiers, or web links for publicly available datasets
- A description of any restrictions on data availability
- For clinical datasets or third party data, please ensure that the statement adheres to our policy

Source data are provided with this paper. The raw reads from Illumina sequencing and Shotgun metagenomic sequencing described in this study, are available at NCBI under the accession no. PRJNA852869 and PRJNA942228, respectively.

Human research participants

Policy information about studies involving human research participants and Sex and Gender in Research.

Reporting on sex and gender	This information has not been collected.
Population characteristics	This information has not been collected.
Recruitment	This information has not been collected.
Ethics oversight	This information has not been collected.

Note that full information on the approval of the study protocol must also be provided in the manuscript.

Field-specific reporting

Please select the one below that is the best fit for your research. If you are not sure, read the appropriate sections before making your selection.

☐ Life sciences ☐ Behavioural & social sciences ☒ Ecological, evolutionary & environmental sciences

For a reference copy of the document with all sections, see nature.com/documents/nr-reporting-summary-flat.pdf

Ecological, evolutionary & environmental sciences study design

All studies must disclose on these points even when the disclosure is negative.

Study description	Here, we conducted a standardized field survey to investigate the relationship of soil properties, the structure and function of the soil microbiome with contrasting plant health outcomes. A total of sixty crop fields were randomly selected across southeast China. Then, we performed microbiome pipelines depending on molecular and cultivable approaches by microcosm experiment and seedling cultivation experiment in the laboratory, and resolved how soil degradation manipulates microbial community, function and thus plant health.
Research sample	In the field survey, we collected 180 composite soil samples (three samples per field) from 60 peanut fields to determine soil physicochemical characteristics, and both bacterial and fungal communities. Meanwhile, we surveyed 9,000 plants (150 plants per field) from 60 peanut fields to determine plant growth and disease severity. As we identified soil pH as a dominant soil factor mediating plant disease severity, the sampled soils were then classified into four categories based on soil pH (i.e., pH 4.0-4.5, 4.5-5.0, 5.0-6.0, and 6.0-7.0). Soil samples falling into the corresponding pH category were adopted as independent replicates, thus the extracted DNA from three soil samples per field was pooled at the site level, resulting in 60 composite DNA samples for Illumina sequencing. Based on the results of the above Illumina sequencing, 12 soil samples that 4 from pH 4.0-4.5, 4 from pH 4.5-5.0 and 4 from pH 5.0-6.0, were selected for shotgun metagenomic sequencing to evaluate the microbial community function. For determining soil suppression on pathogen growth, all soil samples (no. 180) were employed in vitro experiment. Further, we chose 12 soil samples collected from the fields that 3 from pH 4.0-4.5, 3 from pH 4.5-5.0, 3 from pH 5.0-6.0 and 3 from 6.0-7.0, to assess the effects of soil bacterial communities on disease severity in peanut seedling cultivation experiment.
Sampling strategy	In the field survey, we collected paired samples of plants and soils in 60 peanut fields. Three quadrats (replicates, 2 m × 2 m) were arranged in each peanut field, and ten soil cores in each quadrat were pooled and homogenized, resulting in three composite samples per field. Fifty plants of each quadrat were randomly selected to determine disease severity. Thus, our sampling resulted in 180 composite soil samples and 180 plant samples (50 individuals per sample) from 60 peanut fields. Our statistical analyses were based on soil and plant variables across these 180 quadrats, which have been specified in Methods section.
Data collection	The root rot symptoms were assessed by using a scale of 0-4. Soil moisture content (MC) was determined by using the gravimetric method. Soil pH was measured with a glass electrode in a soil-to-water ratio of 1:2.5 (w/v). Soil organic carbon (SOC) was determined by the potassium dichromate oxidation method. Dissolved organic carbon (DOC) was quantified by using a total organic carbon (TOC) analyzer (Multi N/C 3100, Germany). Total nitrogen (TN) was measured by the Kjeldahl digestion method. Ammonium ($NH4+$) and nitrate ($NO3-$) were determined by the UV spectrophotometry method. Total phosphorus (TP) and available phosphorus (AP) were determined by using the molybdenum-blue method with an atomic absorption spectrophotometer. Total potassium (TK) and available potassium (AK) were determined by the flame emission spectrometry method. Soil cation exchange capacity (CEC) was determined through the 1 M ammonium acetate method. Soil texture was determined by the sieve-pipette method. Soil bacterial and fungal communities were sequenced using an Illumina MiSeq PE 250 sequencer (Illumina, USA) at Shanghai Personal Biotechnology Co., Ltd (Shanghai, China). Quantitative real-time PCR (qPCR) was used for the quantification of bacterial and fungal abundance.<br>To assess the responses of root rot pathogen to soils of different pH, plate cultivation experiment containing top and bottom growth areas, and antagonistic experiment were conducted. To further assess the effects of soil bacterial communities on pathogens colonizing in the plant roots, peanut seedling cultivation experiment was conducted. Soil microbial function was analyzed through shotgun metagenomic sequencing on a HiSeq 2500 sequencer (Illumina, USA). Soil microbial volatile compounds were analyzed using GC-Q-TOF QTOF (model Agilent 7890B GC and the Agilent 7200A QTOF, Santa Clara, CA, USA).

Timing and spatial scale	Geographical distribution of sampling peanut fields were from Yichun, Nanchang, Fuzhou, Shangrao, and Yingtan across southeast China. Paired plant and soil sampling was performed in late July 2018, shortly before sprout of root disease. Experiments of laboratory and microcosm were performed from Aug 2018 to Jun 2019.
Data exclusions	All sampled data in field and laboratory experiments were used.
Reproducibility	For Illumina sequencing, DNA PCR amplification was triplicated for each sample using the ABI GeneAmp® 9700 Thermal Cycler. Subsequently, the PCR products were pooled for sequencing analysis on an Illumina MiSeq PE 250 sequencer. For quantitative real-time PCR, each essay was performed in three replicates, and the results were expressed as log10 values (target copy number g-1 soil) for statistical analysis. For other experiments, we set up 3-5 technical replicates for each treatment to ensure the accuracy of the data.
Randomization	Randomization was not relevant in our study as we conducted a field survey to investigate the relationship of soil properties, the structure and function of the soil microbiome with different plant health outcomes.
Blinding	No blinding was applied as no statistical tests were performed where blinding could be applied.

Did the study involve field work? ☒ Yes ☐ No

Field work, collection and transport

Field conditions	The climate of this study area is subtropical monsoon humid. The mean annual precipitation amounts to 1750 mm (average of records over 50 years), and the period of main rainfall is the time between April and June. The monthly average temperature varies from a minimum of 5.9°C in January to a maximum of 30 °C in July.
Location	Field survey was performed across southeast China (28.10-28.90°N, 115.00-116.96°E).
Access & import/export	Soil and plant samples were collected with explicit permission from local and national authorities.
Disturbance	No disturbance was caused by the study.

Reporting for specific materials, systems and methods

We require information from authors about some types of materials, experimental systems and methods used in many studies. Here, indicate whether each material, system or method listed is relevant to your study. If you are not sure if a list item applies to your research, read the appropriate section before selecting a response.

Materials & experimental systems

n/a	Involved in the study
☒	☐ Antibodies
☒	☐ Eukaryotic cell lines
☒	☐ Palaeontology and archaeology
☒	☐ Animals and other organisms
☒	☐ Clinical data
☒	☐ Dual use research of concern

Methods

n/a	Involved in the study
☒	☐ ChIP-seq
☒	☐ Flow cytometry
☒	☐ MRI-based neuroimaging
