# Supplementary material for: Acidification suppresses the natural capacity of soil microbiome to fight pathogenic Fusarium infections
Source: Nat Commun. 2023 Aug 22;14:5090. doi: 10.1038/s41467-023-40810-z (PMC10444831; doi:10.1038/s41467-023-40810-z)
Supplement: Supplementary file 3 — Description of Additional Supplementary Files [file 41467_2023_40810_MOESM3_ESM.pdf]

File Name: Supplementary Data 1

Description: Taxonomic and node properties of the genera distributing in the five predictor phyla based on Random Forest modelling and co-occurrence network analysis.
